# Keratoprosthesis, silicone oil placement, and fluocinolone acetonide implant for treatment of uveitis-associated hypotony and keratopathy

**DOI:** 10.1186/s12348-022-00284-4

**Published:** 2022-02-04

**Authors:** Arman Mosenia, Miel Sundararajan, Jay M. Stewart, Julie M. Schallhorn

**Affiliations:** 1 School of Medicine, University of California San Francisco, 533 Parnassus Ave, San Francisco, CA 94143 USA; 2 Department of Ophthalmology, University of California San Francisco, 490 Illinois Street, San Francisco, CA 94158 USA

## Abstract

**Purpose:**

To describe a case series of combined Boston Type 1 Keratoprosthesis with pars plana vitrectomy, silicone oil placement, and fluocinolone acetonide intravitreal 0.59 mg implant (RETISERT®), and report its safety and efficacy in preventing phthisis bulbi in patients with uveitis-associated hypotony and concurrent corneal edema.

**Findings:**

A retrospective review of patients with chronic uveitis, corneal decompensation and concurrent hypotony who underwent the combined approach described here between 2015 and 2020 was conducted. Three patients were treated using the combined approach. Post-operative recovery was unremarkable in all cases and the patients’ corneal condition remained stable on follow up. No patient developed phthisis, retroprosthetic membrane, or infectious endophthalmitis. Average intraocular pressure one year after intervention was 2.7 to 6.4 mmHg higher compared to a year prior.

**Conclusions:**

The approach described is potentially safe and effective in preventing phthisis and membrane formation in uveitis-associated hypotony and keratopathy.

## Introduction

Hypotony associated with uveitis is an end-stage manifestation of severe ocular inflammation and is associated with loss of visual acuity. It emerges when aqueous production by the ciliary body does not match the outflow [1], and can be present in up to 8% of cases of uveitis [2]. This condition is particularly challenging to treat, and in conjunction with persistent inflammation, can result in phthisis bulbi. Currently, there are no definite treatments for hypotony; however, multiple treatment options have been proposed, including intravitreal injection of viscoelastic material, intraocular gas or silicone oil, topical dopaminergic agonist administration (ibopamine), and pars plana vitrectomy with ciliary membrane removal [3–8].

Long standing hypotony may also cause corneal endothelial pump dysfunction and stromal edema [9]. While the pathophysiology of corneal endothelial decompensation within this context is not well understood, alterations in the composition and flow of aqueous are believed to lead to poor tissue oxygenation and breakdown of the blood-aqueous barrier [10]. Corneal transplantation in the setting of hypotony is technically challenging, and recurrence of edema is common. Consequently, patients often receive multiple corneal transplantations without resolution. Boston Type 1 Keratoprosthesis combined with silicone oil for treatment of concurrent hypotony and corneal edema has shown to improve vision [11]. However, a common complication of this approach is the formation of retroprosthetic membrane [11].

The intravitreal 0.59 mg fluocinolone implant (RETISERT®, Bausch & Lomb, Rochester, NY), is a well described treatment for uveitis, providing excellent control of inflammation for 24 to 30 months after implantation [12]. It exerts its effect locally, and may spare the side effects of systemic immunosuppression in some patients [13]. Although it has been associated with a significant risk of developing ocular hypertension and glaucoma [14], it has not been found to improve intraocular pressure when implanted in hypotonus eyes [15].

The purpose of this study is to report the authors’ experience in treating corneal edema in the setting of uveitis-associated hypotony with combined Boston Type 1 Keratoprosthesis placement (Massachusetts Eye and Ear, Boston, MA), silicone oil injection, and 0.59 mg fluocinolone acetonide intravitreal implant**.**

## Materials and methods

This is a retrospective study of patients with hypotony who underwent fluocinolone acetonide implantation and single-stage keratoprosthesis with silicone oil injection between 2015 and 2020. Patients with chronic uveitis who had evidence of corneal decompensation with concurrent hypotony were included, and their electronic health records were reviewed.All patients underwent surgery for an indication of corneal edema in the setting of uveitis and hypotony, and all patients elected to undergo fluocinolone acetonide implantation for uveitis control. All patients underwent 23-gauge pars plana vitrectomy with silicone oil injection (5000 cSt). One patient had a pre-existing retinal detachment. Intraocular pressures after keratoprosthesis placement were estimated by scleral pressure and were measured using pneumatonometry in the inferotemporal quadrant to compare with corneal pressure measurements prior to keratoprosthesis placement [16].

## Case presentations

### Case 1

A 47-year-old woman with a history of chronic unilateral non-granulomatous anterior uveitis with cystoid macular edema (CME) of the left eye presented with eye pain for surgical visual rehabilitation evaluation (Table 1). Her history was complicated by non-clearing vitreous hemorrhage due to neovascularization of the pars plana, and pseudophakic bullous keratopathy. She had previously undergone Descemet’s stripping endothelial keratoplasty (DSAEK) followed by penetrating keratoplasty (PK), both of which were complicated by graft failure. Therefore, she was treated with pars plana vitrectomy, silicone oil placement, and fluocinolone acetonide intravitreal implant insertion with repeat PK. Six months after this surgery, she underwent silicone oil removal, but developed hypotony with choroidal detachment and an overlying retinal detachment. Due to persistently low intraocular pressure, a combined aphakic keratoprosthesis with retinal detachment repair and silicone oil injection was carried out. Visual acuity prior to surgery was light perception (LP) and intraocular pressure was 4 mmHg. The retinal detachment was noted to be complex, and the vision became no light perception (NLP) after repair. The retina has remained attached at one-year follow up and the patient has maintained a stable keratoprosthesis without bandage contact lens placement.

**Table 1:** Clinical profile of patients

Case	Age/ Sex	Uveitis class	Chief complaint	Previous Transplants	Vision, Before	Vision, After	Complications	Follow Up (years)
1	47/F	Anterior, unilateral	Pain, visual rehabilitation	3	LP	NLP	None	1
2	53/F	Panuveitis, bilateral	Corneal edema	0	LP	20/800	None	1
3	57/M	Panuveitis, bilateral	Declining vision	2	HM	20/125	None	5.5

*LP* Light perception, *NLP* No light perception, *HM* Hand motion

### Case 2

A 53-year-old woman with bilateral steroid response glaucoma and chronic panuveitis presented for surgical treatment of corneal edema and hypotony (Table 1). Her left eye was noted to be hypotonus with diffuse corneal edema. Visual acuity in the left eye was light perception (LP), and intraocular pressure was 1 mmHg at the time of initial evaluation. She was offered a single stage combined fluocinolone acetonide implantation with an aphakic keratoprosthesis and silicone oil insertion for visual rehabilitation. The operation was completed without complication, with a resultant BCVA of 20/800 and an intraocular pressure of 13 mmHg. Bandage contact lens fitting proved difficult due to the unusual ocular surface contour, but the ocular surface has remained stable without a bandage contact lens placement. The patient has not developed a retroprosthetic membrane at one-year follow up.

### Case 3

A 57-year-old man with a history of bilateral immune reconstitution panuveitis secondary to CMV retinitis in the setting of human immunodeficiency virus (HIV) developed significant hypotony in the right eye requiring repeat injections of hyaluronic acid (Table 1). The left eye had no light perception. Vision in the affected eye was hand motions. He had previously undergone three vitrectomies and two keratoplasties. The keratoplasties had presumably failed due to hypotony. He developed an inferior retinal detachment, and a combined surgical approach was pursued for aphakic keratoprosthesis placement, removal of existing intraocular lens, pars plana vitrectomy and silicone oil injection. He had a fluocinolone acetonide implant, which was most recently exchange a year prior to the surgery. Post-operative recovery was unremarkable. At one-year follow up, best corrected visual acuity was 20/125. He has subsequently undergone multiple dexamethasone intravitreal steroid implant (OZURDEX®, Allergan, Irvine, CA) injections and four supplemental intravitreal silicone oil injections to increase the intraocular pressure.

He has been maintained without bandage contact lens placement, as achieving an appropriate fit was difficult. The ocular surface was stable, and he did not have a retroprosthetic membrane for 5 years after surgery. However, he developed a small epithelial defect at 5 years that responded within 1 week to amniotic membrane placement.

## Discussion

Hypotony may lead into several irreversible, vision-impairing complications in the posterior segment. The resulting shrinkage and collapse of the scleral wall due to low intraocular pressure (IOP) is thought to fold and wrinkle the chorio-retina, particularly in the peri-foveal region, distorting neurosensory elements with permanent visual loss [1]. Additionally, the malformation of the globe may change retinal capillary permeability, resulting in cystoid macular edema as seen in all three cases here. Therefore, early detection of hypotony and its management dictates final visual outcomes in these patients. In all three cases, the average pressure in the affected eye was higher after keratoprosthesis insertion compared to a year prior (Table 2). In one of three, silicone oil was injected at least once in the year prior to surgery. While average IOP one month prior to and following silicone oil insertion were nearly identical at 6 mmHg (*n* = 2) and 6.7 mmHg (*n* = 3), respectively, average IOP before and after silicone oil placement with keratoprosthesis was 4 mmHg (*n* = 1) and 10 mmHg (*n* = 3). These findings suggest that combined keratoprosthesis insertion and silicone oil placement may help maintain higher IOP when compared to silicone oil alone, thus lowering the probability of hypotony maculopathy.

**Table 2:** Average IOP within one year before and after combined keratoprosthesis and silicone oil insertion

Case	Pre-Intervention IOP	Post-Intervention IOP	*P*-value < 0.05
1	6.07 [4.79 7.35]	12.50 [10.32 14.68]	*
2	8.70 [3.74 13.66]	11.40 [10.47 12.33]	
3	6.93 [5.93 7.95]	10.45 [7.49 13.43]	*

Numbers in brackets are confidence intervals of the mean pressures. All pressured reported in mmHg. *P* value corresponds to the difference between pre-intervention and post-intervention mean pressures

In phthisical eyes, the corneal epithelium is often normal, and the ensuing edema is stromal due to endothelial decompensation [9]. This has been shown to be reversible by addressing the underlying cause of hypotony, in trabeculectomy for example [17]. Nevertheless, uveitis-induced hypotony is challenging to treat and often persists. Therefore, the main treatment option is keratoplasty (DSAEK or PK), which has a dim chance of success without addressing the underlying cause and improving IOP. Two of the three cases reported here had received corneal transplants, all of which had failed. Therefore, permanent keratoprosthesis may improve vision and help minimize the number of surgical interventions, especially in those with recurring graft failure.

Retroprosthetic membrane formation is a common occurrence after keratoprosthesis surgery [18]. In a prior study describing combined keratoprosthesis surgery and silicone oil insertion for treatment of hypotony secondary to multiple etiologies, retroprosthetic membrane was seen in 54% of patients [11]. In those with uveitis, however, this can be more prevalent due to increased inflammation, affecting visual acuity and predisposing them to sterile keratolysis [19]. Notably, none of the cases reported here developed retroprosthetic membranes. The presence of the intravitreal fluocinolone implant likely contributed to this, as local high dose steroids have been associated with decreased vascular permeability and fibroblast proliferation, and have been shown to lengthen the time to retroprosthetic membrane development [18]. Specifically, the fluocinolone acetonide implant has been associated with improved long-term outcomes with glaucoma drainage device placement [20].

All eyes in this study also had stable ocular surfaces without the need for a bandage contact lens. The patients in this study had difficulty getting a lens fit owing to the abnormal contour of their ocular surface in the setting of a pre-phthisical state prior to keratoprosthesis placement. Even extremely flat, large diameter lenses were unable to be retained successfully. Despite this, these patients did not experience epithelial breakdown and keratolysis. Stability of the ocular surface in the setting of silicone oil and keratoprosthesis placement to treat hypotony has previously been noted [11, 21]. We theorize that shrinkage of the eye due to the pre-phthisical state prior to surgery affords better protection of the ocular surface from the eyelids, and thus enables more stability of the epithelium.

This study has several strengths, including unremarkable post-operative recovery and absence of any complications, particularly retroprosthetic membrane formation, infectious endophthalmitis and sterile keratolysis in uveitis-associated hypotony and keratopathy. While inferotemporal scleral pneumatonometry is a promising method for measuring IOP in those with keratoprosthesis, this has not been validated in eyes with hypotony [16]. Future work can focus on further characterizing the impact of this intervention on IOP. Regardless, the combined approach was clinically effective in preventing phthisis for at least one year after surgery in these patients.

## Conclusion

Combined treatment of uveitis-associated hypotony and keratopathy with Boston Type 1 Keratoprosthesis, silicone oil placement, and fluocinolone acetonide implant may be a safe and effective method to prevent phthisis.

## Data Availability

The data used and analyzed during the current study are available from the corresponding author on reasonable request.

## Abbreviations

IOP: Intraocular pressure
LP: Light perception
NLP: No light perception
HM: Hand motion
